# Surgical Residents´ Results Seem to be Non-Inferior Comparing to More Experienced Surgeons in Femoral Neck Fracture Osteosynthesis

**DOI:** 10.1007/s43465-023-00992-6

**Published:** 2023-10-03

**Authors:** Nora Forsbacka, Terhi Kolari, Marjo Talme, Ville Bister

**Affiliations:** 1 Turku University Hospital Trauma Unit, Turku University Hospital, Turku, Finland; 2 Department of Biostatistics, University of Turku, Turku, Finland; 3 Helsinki University Hospital, Hyvinkää Hospital, Hyvinkää, Finland; 4 Helsinki University Hospital Trauma Unit, Töölö Hospital, Helsinki, Finland; 5 Department of Surgery, Clinicum, Faculty of Medicine, University of Helsinki, Helsinki, Finland; 6 Peijas Hospital, Vantaa, Finland

**Keywords:** Femoral neck fracture, Osteosynthesis, Surgical experience, Surgical training

## Abstract

**Purpose of the Study:**

Femoral neck fracture osteosynthesis is usually performed by using dynamic hip screw (DHS) or multiple parallel cannulated screws (MCS). In orthopedic surgery training, certain hip fractures are one of the most common operations performed by residents. It has been questioned, whether residents can provide as safe treatment and patient outcomes as those of more experienced surgeons. The aim of this study was to evaluate the effect of surgical experience on risk of complications by comparing the surgical performance and clinical outcomes in femoral neck osteosyntheses between surgical residents and orthopaedic surgeons.

**Methods:**

All patients with femoral neck fracture admitted to Helsinki and Uusimaa Hospital District (HUS) Hyvinkää Hospital from 2011 to 2016 were collected as research material. 88 hip fractures (87 patients) treated with DHS or MCS osteosyntheses were included in this study. The patients were divided into 2 groups, based on the surgeon’s experience: an orthopedic surgeon group (n = 68) and a surgical resident group (n = 20). All data from complications, reoperations, and the duration of operations were collected.

**Results:**

There was no significant difference in characteristics of operated patients between orthopaedics and residents group. There was no significant difference in complications or re-operations between groups (p = 0.4, p = 0.2). Surgical residents had statistically longer surgical time (mean 76 min, 95% CI 62.92 min, mean 46 min, 95% Cl 42.51 min; p-value < 0.001). Still surgical time was not a risk factor for complication (p-value 0.5).

**Conclusion:**

Our results show that surgical residents´ outcomes in femoral neck fracture osteosynthesis seem to be as favorable as those of orthopedic surgeons; the operations just last slightly longer.

## Introduction

Osteosynthesis is used with certain types of femoral neck fracture. Patients are typically younger with undisplaced fractures, while retaining the patient’s own hip joint is a priority [1]. Osteosynthesis, also known as internal fixation, is usually performed with either dynamic hip screws (DHS) or multiple parallel cannulated screws (MCS). Multiple-screw fixation is less invasive and retains more viable bone [2, 3] than does DHS fixation, which appears to be biomechanically more stable [4–6]. The optimal treatment for intracapsular hip fracture has been controversial [7–9].

Operative treatment of hip fracture is one of the most common procedures during orthopedic surgery training. Residents are also vital to the clinical workforce in hospitals and provide a large portion of the daily patient care. Surgical training aims to prepare residents to provide high-quality care, and it requires appropriate amounts of hands-on experience in the operating room. Whether residents can provide treatment as safe and patient outcomes as favorable as those of more experienced surgeons has been questioned.

To evaluate the risks and quality of treatment, researchers have defined learning curves from various surgical procedures. These describe either the minimum time of training or number of surgeries a surgical trainee needs to be able to achieve the required quality of treatment and minimal risk for complications. Van der Leeuw et al. [10] concluded in a systematic review including nearly 100 studies that patient care appears to be safe and equal in quality when delivered by residents. Of course, complications can still appear regardless of the experience of the surgeon performing the operation.

Femoral neck fracture operations include risks of complications, such as infections and surgical site hematomas. Femoral neck fractures, treated with osteosynthesis, can sometimes cause non-union, implant failure, bone misalignment or femoral head avascular necrosis (AVN) [1, 11]. Rare complications of surgical treatment include sciatic nerve injuries and iatrogenic fractures.

In the review by van der Leeuw et al. [10] examining treatment outcomes with residents, only 7 studies were of orthopedic residents; only one of these studies covered hip fractures [12]. The study examined whether the complication and mortality rates for hip fracture patients were higher with less experienced residents and included both femoral neck fractures and intertrochanteric fractures in the analysis. There was no mention about the surgical treatment used. The overall mortality rate was slightly higher in teaching than in nonteaching hospitals (3.7% vs. 3.6%). In contrast, the complication rates showed no differences between teaching and nonteaching hospitals.

To our knowledge, no studies have evaluated the impact of surgical expertise on the outcomes of femoral neck fracture osteosynthesis. The aim of this study was to evaluate the effect of surgical experience on the risk of complications by comparing the surgical performance and clinical outcomes between surgical residents and orthopedic surgeons.

## Materials and Methods

This was a retrospective single center study, conducted at Helsinki and Uusimaa Hospital District (HUS) Hyvinkää Hospital, Finland. HUS Hyvinkää Hospital is a public hospital (Level III trauma center) providing 24/7 traumatological treatment to a population of 190,000, which is approximately 4% of the population in Finland.

Patients with hip fracture treated with osteosynthesis were selected for the study from the period January 1st 2011 to December 31st 2016. This included 361 patients, of which 87 were treated with DHS or MCS osteosynthesis and had an International Classification of Diseases (ICD-10) S72.0 femoral neck fracture diagnosis (exclusion criteria are seen in Table 1). One patient had both hips operated (two different injuries), therefore the study included total of 88 operated femoral neck fractures.

**Table 1:** Exclusion criteria

Exclusion criteria	n
Total	361
Pertrochanteric	109
Other osteosynthesis or arthroplasty	154
Died before operation	2
Non-operative treatment	1
Re-operation	2
Age under 16 yrs	4
Treatment in other hospital	1
Primary x-rays not available	1
Included patients	**87**
Included hip fractures	88*

*One patient had both hips operated (two different injuries)

The surgical information program Centricity Opera provided data about the surgeon and duration of the surgery. The patients were divided into 2 groups: surgeries performed by orthopedic surgeons (n = 68) and those performed by residents (n = 20). Both orthopedic and resident groups had 9 different surgeons (total of 18). The number of operations per surgeon in this study was on median 8.5 (range 2 to 13) in the orthopedic group and on median 2 (range 1 to 5) in the resident group. Residents operated 15 hips independently and 5 hips under senior supervision but also in those cases the resident was the primary surgeon. Due to very small amount of operations under supervision, we decided to include these 5 operations to the resident group as a whole. Regarding this study, previous experience of these operations (DHS or MCS osteosynthesis) is approximately over 10–20 in the orthopedic group and 0–5 in the resident group.

Tobacco and alcohol consumption were also recorded. The patient was recorded as a heavy user if there was a history of several hospital visits for alcohol intoxication.

Postoperative recordings and X-rays were used to determine the implementation and success of the treatment, as well as possible complications. The complications included infections requiring hospitalization, hematomas of the surgical site requiring reoperation, and AVN changes. Fixation material failures were included as complications. If a fracture gap was still visible in the control x-rays after a 6-month follow-up period, it was considered a non-ossification and included as a complication.

Possible reoperations due to complications or treatment failure were recorded. These included also osteosynthesis conversion to arthroplasty. The follow-up period was 2–5 years.

### Statistical Analysis

The clinical characteristics were summarized with counts (n). The associations between the categorical data were analyzed with Fisher’s exact test. Survival analysis was performed to investigate complication and the need for reoperation. The patients were allocated into groups by the surgeon performing the operation.

First, univariate analyses for the cumulative percentages of complication and reoperation need were estimated, using the Kaplan–Meier technique, and the differences between the orthopedic and resident groups were tested, using the log-rank test. Second, more complicated models with multiple factors were performed with Cox’s proportional hazard models. Differences between the orthopedic and resident groups were quantified by calculating hazard ratios (HRs) with 95% confidence intervals (95% CIs), using Cox’s regression models. The validity of proportional hazards assumption was assessed both visually and numerically, and no marked deviations for assumptions were observed. The initial model included the surgeon performing the operation, osteosynthesis method, tobacco, alcohol, patient age, gender, and Garden classification; nonsignificant factors were gradually omitted.

The operation times between the operator groups were compared with one-way analysis of variance (ANOVA). All tests were performed as two-sided with a significance level set at 0.05. The statistical computations were performed, using SAS Systems for Windows, Version 9.4 (SAS Institute Inc., Cary, NC, USA).

## Results

The majority of patients were female, and the mean age of the whole study population was 69.7 (sd 17.1) years. Sex or patient age showed no significant differences between groups (p-values 0.8, 0.9). 44 patients used tobacco and 15 were heavy users of alcohol. There were no significant differences in patient intoxicant use between the orthopedic and resident groups (p-values 0.6, 0.7). If a patient used alcohol, the same patient was also more prone to use tobacco (p-value < 0.001). The clinical characteristics are shown in Table 2. There were no significant differences in Garden classification or anatomical fracture types between the operating groups (p-value 0.3 and 0.8).

**Table 2:** Clinical characteristic

Clinical characteristics	Total	OS	Rs	p-value
All patients (n)	87	67	20	
Female (n)	53	40	13	0.8
Age (mean)	69.7	69.8	69.2	0.9
Tobacco (n)				0.6
No	43	35	8	
Yes	22	16	6	
N/A	22	16	6	
Alcohol (n)				0.7
No	32	24	8	
Yes	15	13	2	
N/A	40	30	10	
Smoking + alcohol combination (n)				1.0
No	24	19	5	
Yes	9	7	2	

*OS* Orthopedic surgeons, *Rs* Residents, *N/A* not applicable

In all, 68 hips were operated by the orthopedic surgeon group and 20 the resident group; 47 hips were treated with a DHS and 41 with screws. The plate sliding screw, i.e. the DHS group, also included patients who had a single antirotation screw installed in addition to the plate sliding screw. The orthopedic surgeon group had 35 DHS and 33 MCS osteosyntheses, while the resident group had 12 DHS and 8 MCS fixations. There were no significant differences between the groups by used osteosyntheses (p-value 0.4).

In all, 22 patients had some complications, of which 19 required reoperations. In the orthopedic group, 19 patients had complications and 17 patients were reoperated. In the resident group, 3 patients had complications and 2 had to be reoperated. All of the postoperative x-rays were accepted by senior surgeons and their colleagues during the primary ward treatment period, and there were no early reoperations because of poor reduction or suboptimal fixation. There were no statistically significant differences in complications or reoperations between the groups (p-values 0.4, 0.15).

Significant differences were observed in the duration of surgeries. The operating mean time in the orthopedic group was 46 min (95% CI 42,51 min) and in the resident group 76 min (95% CI 62,92 min) (p-value < 0.001). Duration of surgery was not a risk factor for complication or reoperation (p-value 0.3).

Alcohol consumption was a risk factor for reoperation (log-rank test p-value = 0.03, Cox’s proportional hazard HR = 2.64, 95% CI 0.96, 7.27, p-value 0.06). However, the analyses of tobacco and alcohol use were limited due to missing data. Of the 33 patients having information on their intoxicant and tobacco use, 7 of those 9 who used both tobacco and alcohol had complications and/or reoperation, while 12 of 24 nonusers had complications and/or reoperation (p-value 0.24).

The other clinical characteristics (patient age, gender, and Garden classification) were not statistically associated with complication or reoperation (p-values 0.1, 0.9, 0.1).

Overall, there were no infections requiring hospitalization in either of the osteosynthesis groups. In the orthopedic group 8 and in the resident group 1 fracture remained unossified. At follow-up, fixation failed in 7 patients, all of whom were in the orthopedic group, while 2 cases of AVN developed in the orthopedic group and 1 in the resident group. Both groups had one for other complications, see Table 3.

**Table 3:** Surgical expertise

Surgical expertise	OS	Rs	p-value
Osteosynthesis (n)	68	20	
DHS	35	12	0.4
MCS	33	8	0.4
Duration of surgery, mean (min)	46	76	< 0.001
Complication (combination)	19 (30%)	3 (15%)	0.4
Infection	0	0	
Non-union	8 (12%)	1 (5%)	
Fixation failure*	7 (12%)	0	
AVN	2 (3%)	1 (5%)	
Other**	2 (3%)	1 (5%)	
Reoperation	17 (25%)	2 (13%)	0.15

*OS* Orthopedic surgeon, *Rs* Resident, *DHS* dynamic hip screws, *MCS* multiple parallel cannulated screws, *AVN* avascular necrosis

*Fixation material such as screws or collum blade movement in follow-up

**Surgical site hematoma, delayed ossification, long-term pain

## Discussion

Femoral neck fractures treated with osteosyntheses such as DHS or MCS are relatively small surgical procedures but have risk of reoperation due to complications such as non-union, mechanical failure, or AVN [1, 11, 13]. Although MCS fixation may have some benefits compared with DHS, it also seems to have a higher risk of conversion to hemi/total hip arthroplasty [14]. Recently, it has also been suggested that elderly patients may benefit from primary hemiarthroplasty in comparison to those using MCS when improved mobility and risk for reoperations are considered [15].

Postoperative infections range from 0 to 10% after internal fixation of femoral neck fracture [2]. In our study, there were no infections leading to hospitalization or even emergency department/out-patient clinic visits. Minor wound problems or superficial wound infections treated at the primary health care level may possibly have been present without the need for surgical intervention. Our results showed slightly higher rates of non-union and AVN than previously reported [14]. There were 4 patients who had problems with recovery (malunion or non-union), but never went through a reoperation because of their poor overall condition. In more complex surgeries there is a learning curve with both complications and surgical time [16–19]. Low number of repetitive procedures has led to consider different training methods like virtual reality [20].

Quality improvement efforts in surgery have largely focused on reducing the risk of complications after surgical procedures. Whether residents can provide as safe treatment and patient outcomes as favourable as those from more experienced surgeons has often been questioned. Sometimes the longer surgical time may take the glory away from successful outcomes. In our study, the surgical time was longer in the residents’ group, as expected. More experienced surgeons operate faster than early-state residents [16, 21], and there is also a learning curve up to at least 20 to 30 operations when considering the duration of surgery [19, 22]. However, perhaps the most important finding was that this increase in surgical time seemed not to be associated with more unfavourable patient outcomes. The same conclusion was also previously made with intertrochanteric nailing with simple fracture patterns [23]. Surgical time can impact the effectiveness and costs of surgery, but without surgical training, there will be no future experienced surgeons. Longer surgical times can also affect the complication rates, including postoperative infections, when approximately 2 h is exceeded [24]. With femoral neck fracture osteosynthesis it is rarely a problem, since the surgical time ranges from approximately 30 min to 90 min [25]. In our study, residents performed 20 (23%) of these femoral neck osteosynthesis operations, which is already slightly higher than the reported amounts in Norway (13.5%) [25]. We must still bear in mind that not all surgeries are suitable for inexperienced surgeons. Displaced fracture patterns and more seldom-performed operations may lead to poorer results [26].

Our patients were younger on average (69.7 years) than in previously reported hip osteosynthesis studies [14, 25] so the results of complication rates are not directly comparable. Although we had limited data on alcohol and tobacco consumption, the results are leaning towards a larger material studied in general surgery revealing that combined consumption is more likely to lead to complications, readmissions, and reoperations [27].

This study has strengths and some limitations. This was a single center study of all operated femoral neck fracture patients in HUS Hyvinkää Hospital from the selected period of 6 years. The teaching methods and unit traditions stayed the same, and patient chart review could be done in detail. The research design was retrospective and the number of operated fractures (n = 88) was relatively limited. When making further conclusions, one has to bear in mind that the research population should have been larger. In this case, it would have needed either longer period of time for data collection or data combination with another hospital. We must also acknowledge that surgical training variates around the world and surgical residents’ operating results may also vary.

We evaluated the effect of a surgeon’s expertise on the outcome and safety of femoral neck fractures treated with osteosynthesis. Considering the equal complication profile in this study, it may be possible to offer residents further opportunities to perform the operation. Our results show that femoral neck fracture osteosynthesis seems to be non-inferior in the hands of surgical residents.
